# Respiratory and bloodstream coinfections and antimicrobial use in hospitalised patients with moderate to severe COVID-19: An Australian retrospective cohort study

**DOI:** 10.1371/journal.pone.0352344

**Published:** 2026-07-07

**Authors:** Laura Frederiksen, Shradha Subedi, Keat Choong, James Anderson, Timothy Baird

**Affiliations:** 1 Department of Respiratory Medicine, Sunshine Coast Health, Queensland, Australia; 2 School of Medicine and Dentistry, Griffith University, Gold Coast, Queensland, Australia; 3 Faculty of Medicine, The University of Queensland, Brisbane, Queensland, Australia; 4 Department of Infectious Diseases, Sunshine Coast Health, Queensland, Australia; 5 Sunshine Coast Health Institute, Sunshine Coast, Queensland, Australia; 6 Infection Research Network Sunshine Coast, Sunshine Coast Health, Queensland, Australia; 7 University of the Sunshine Coast, Sunshine Coast, Queensland, Australia; Aga Khan University, PAKISTAN

## Abstract

**Background:**

COVID-19 remains a leading infectious cause of death and hospitalisation globally. Coinfections with SARS-CoV-2 and other respiratory pathogens may result in more severe illness, however the prevalence of coinfection in Australia is unknown.

**Aims:**

This Australian study aimed to determine the prevalence and microbiology of respiratory and bloodstream coinfections, antimicrobial use, and outcomes in hospitalised patients with moderate to severe COVID-19.

**Methods:**

This was a retrospective cohort study of adult patients with moderate to severe COVID-19, admitted at the Sunshine Coast University Hospital from February to July 2022. Data regarding patient characteristics, comorbidities, microbiological results, hospital length of stay, intensive care unit admission, and mortality were compared between the coinfection and no-coinfection groups. Logistic regression analysis was performed to identify factors associated with coinfection.

**Results:**

Coinfection was documented in 23 (12%) of the 190 patients admitted with moderate-severe COVID-19. Bacterial infections were the most common (54% of coinfection episodes), followed by fungal (32%), and viral (14%). Antibiotics were prescribed for 74% of patients, for a median duration of 6 days (IQR 4–8 days). Patients with coinfection had a median length of stay of 9 days (IQR: 4–19.5) compared to 6 days in the no-coinfection group (IQR: 3–9; *p* = 0.047). There was no mortality difference between the two groups. Patients admitted to intensive care had higher odds of coinfection compared to patients not admitted to intensive care (OR 3.39, 95% CI 1.19–9.66, *p* = 0.02). Severe COVID-19 and Aboriginal and Torres Strait Islander descent were also associated with coinfection. The causal nature of these relationships requires further interrogation.

**Conclusions:**

The prevalence of respiratory and bloodstream coinfection was low in our cohort of hospitalised COVID-19 patients. Despite non-standardised microbiological testing, antibiotic use was disproportionately high. Further work is required to define risk factors and improve diagnosis of COVID-19-associated coinfection, to better inform antimicrobial stewardship.

## Introduction

The coronavirus disease 2019 (COVID-19), caused by the severe acute respiratory syndrome coronavirus 2 (SARS-CoV-2), remains a leading infectious cause of death and hospitalisation worldwide [1]. Disease severity in COVID-19 can be influenced by simultaneous or secondary respiratory infections with other pathogens; so-called coinfections and superinfections [2]. Coinfections with other viruses, bacteria and fungi can worsen disease severity through immune overload, and complicate clinical management [3]. Improved awareness, diagnostic techniques and treatments are required to reduce the risk of severe complications and mortality [3].

According to the US Centre for Disease Control, coinfection is defined as an infection which occurs at the same time the initial illness, while a secondary or superinfection occurs after the initial infection [4]. Despite these definitions, the terms coinfection, secondary infection and superinfection are often used interchangeably in the medical literature [4]. Infections may also be categorised as community-acquired (within 48 hours of admission) or hospital-acquired (beyond 48 hours); which are the definitions that will be used in this manuscript [4].

Published data suggests that the prevalence of respiratory coinfection in COVID-19 is highly variable, depending on the population of interest. Community-acquired infection rates vary from 2% to 20%, with common pathogens including *Streptococcus pneumoniae*, *Staphylococcus aureus*, *Haemophilus influenzae*, *Klebsiella pneumoniae,* Influenza A and B, and Respiratory Syncytial Virus (RSV) [2,5–13]. Hospital-acquired infection rates range from 3% to 45% and are predictably highest in intensive care unit (ICU) patients [2,7–11]. *S. aureus, Pseudomonas aeruginosa, Stenotrophomonas maltophilia, K. pneumoniae, and Aspergillus* are common hospital-acquired respiratory pathogens [2,5–11,14,15].

Despite generally low rates of bacterial coinfection, antibiotic use during the COVID-19 pandemic was widespread. Reported frequencies of antibiotic prescription range from 35% to 98%, for cohorts where the identified rate of bacterial co- or secondary infection was as low as 3.5% to 8% [5–8]. As an unwelcome consequence of the COVID-19 pandemic there has been a rapid increase in multi-drug resistant organisms, and inappropriate antimicrobial use may be one causative factor [16]. The need for evidence-based antibiotic prescribing guidelines is apparent but is limited by our understanding of risk factors and imprecise diagnostic tools [9,17].

Compared to patients with isolated SARS-CoV-2 infection, those with coinfections or secondary infections experienced higher mortality, ICU admission, and hospital length of stay (LOS) in some studies [2,7,10,17]. Early identification of patients with coinfection may aid in reducing adverse outcomes, but has proven challenging due to a lack of established risk factors, and imperfect diagnostic tests for coinfection [17,18]. Increasing age, chronic renal disease, COVID-19 severity, mechanical ventilation, and leukocyte count are risk factors that have been identified in a small number of publications, but many studies have failed to demonstrate statistical significance [2,7,8,10].

While coinfection and secondary infection in COVID-19 have been described internationally, there is very little published data on the Australian experience, largely due to the delayed spread of SARS-CoV-2. This single-centre study aimed to determine the local prevalence and microbiology of respiratory and bloodstream coinfections, risk factors for coinfection, antimicrobial use, and outcomes in hospitalised patients with moderate to severe COVID-19.

## Methods

### Study design and patient population

We conducted a retrospective observational cohort study of adult patients admitted to the Sunshine Coast University Hospital with COVID-19 from February to July 2022. A patient list was obtained using the electronic medical record to retrieve all inpatient admissions where COVID-19 was listed as a diagnostic code. Medical records were accessed for data collection between 1^st^ October 2022 and 1^st^ March 2023. All data were deidentified after accessing the medical record and prior to analysis.

**Fig 1:**
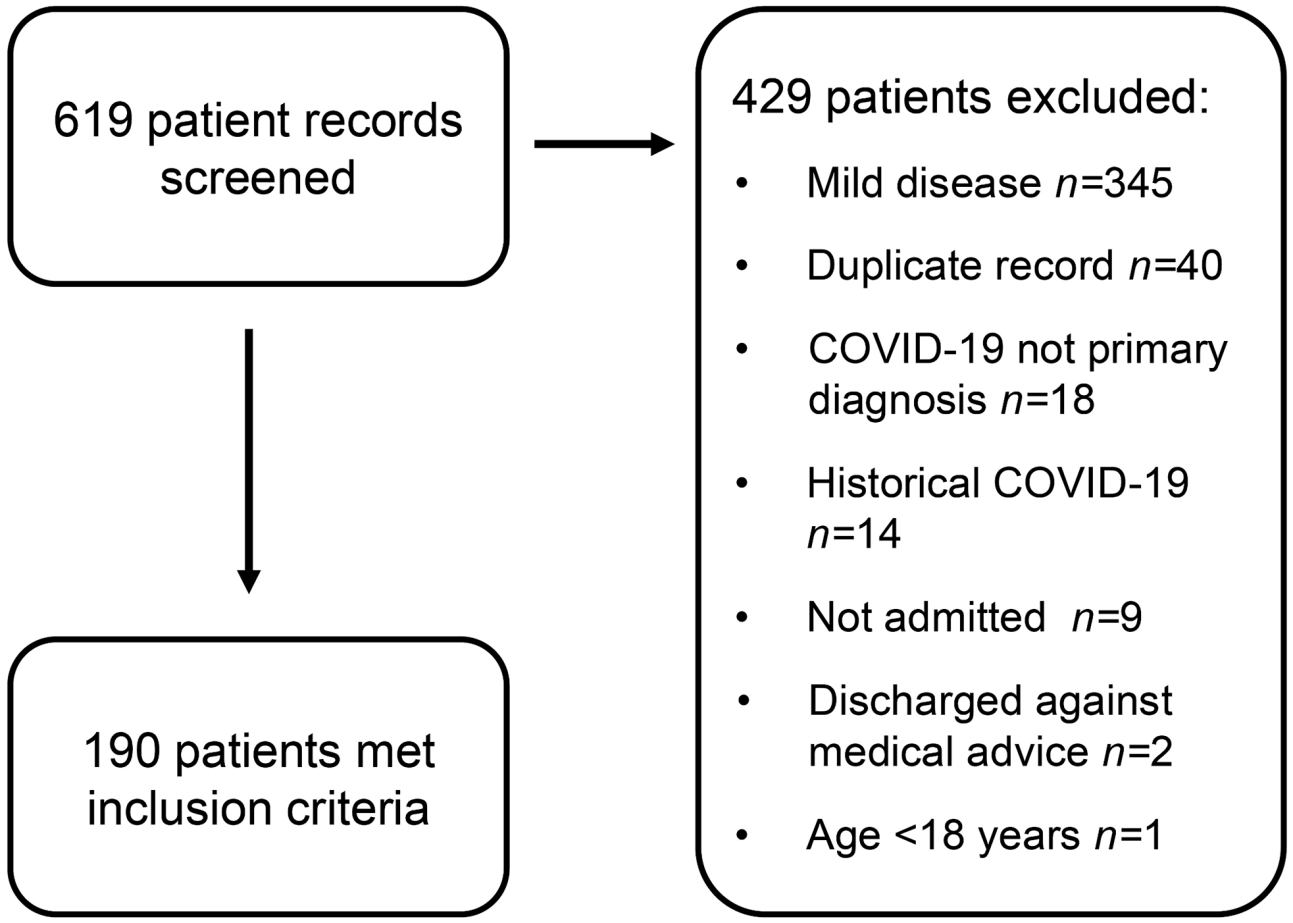
Flow diagram of patient inclusion and exclusion criteria.

Patients were included in the study if they were at least 18 years of age and had moderate or severe COVID-19 as the principal diagnosis, as defined by the Australian National Clinical Evidence Taskforce COVID-19 guidelines (Table 1) [19]. Disease severity was taken as the worst severity during the admission. The diagnosis of COVID-19 could be made by nasopharyngeal polymerase chain reaction (PCR) or rapid antigen testing. Patients were excluded if they had mild or incidental COVID-19, historical COVID-19, duplicate records, discharged against medical advice, or were less than 18 years old (Fig 1). Patient data relating to demographics, comorbidities, pathology, microbiology, radiology, and antimicrobial use were collected from the electronic medical record. This study aimed to identify respiratory or bloodstream coinfections, and so microbiological results relating to other infections were not routinely collected. Outcomes included ICU admission, hospital LOS, and all-cause mortality (30- and 90-day). Mortality statistics were based upon hospital records and out of hospital linkage data.

**Table 1:** Definition of disease severity for adults, as described by the National Clinical Evidence Taskforce for COVID-19 Australian Guidelines [19].

**Mild illness**	A patient who is asymptomatic or has mild symptoms and signs • No new shortness of breath • No evidence of lower respiratory tract disease
**Moderate illness**	A stable patient with lower respiratory tract disease evidenced by: • Oxygen saturation 92–94% on room air at rest, or • Desaturation or breathlessness on exertion, or • On imaging
**Severe illness**	A patient with moderate disease who is deteriorating, or a patient meeting any of the below criteria: • respiratory rate ≥ 30 breaths/min • oxygen saturation < 92% on room air at rest or requiring oxygen • lung infiltrates > 50% on imaging

### Definitions

Coinfection was defined as the positive culture of a bacterium or fungus from sputum, blood, bronchoalveolar lavage or endotracheal aspirate, a positive respiratory viral PCR on nasopharyngeal swab, a positive *Pneumocystis jirovecii* PCR on induced sputum or bronchoalveolar lavage, or a positive *Pneumococcal* or *Legionella* urinary antigen test. This definition may capture infection and colonisation, and so treatment data for each isolate was recorded for later analysis. Blood cultures growing typical skin contaminants (such as coagulase-negative Staphylococci) were excluded [20]. All laboratory and microbiological tests were performed according to the clinical needs of the patient, at the discretion of the treating physician. Coinfections were categorised as community-acquired (occurring ≤2 days from hospital admission); or hospital-acquired (>2 days after admission). Radiologic assessments included chest radiograph or computed tomography. Radiologic abnormalities were defined as consolidation, patchy opacification, interstitial abnormality, or ground glass opacity. The presence of a radiologic abnormality was determined on the basis of the radiology report in the medical record.

Comorbidities and immunocompromise were defined as per published guidelines from the Australian Technical Advisory Group on Immunisation and the National Clinical Evidence Taskforce for COVID-19 [19,21]. Immunocompromise included immunodeficiency (e.g., haematological neoplasm, solid organ transplant, haematopoietic stem cell transplant (within 24 months), primary immunodeficiency or advanced/untreated HIV) and/or immunosuppressive therapy (e.g., chemotherapy, whole body radiotherapy or total lymphoid irradiation, high-dose corticosteroids (≥20 mg prednisone per day, or equivalent, for ≥14 days), and selected other potent immunosuppressive therapies) [19].

### Statistical analysis

Descriptive statistics were used to explore patient characteristics, microbiological results, antimicrobial use, and patient outcomes. Due to the presence of skewed or small-sample size data, comparison between groups was made using Wilcoxon’s rank-sum test (for continuous variables), and Fisher’s exact test (for categorical variables). To identify factors associated with coinfection, variables hypothesised to potentially be associated were assessed using univariate logistic regression (Table 5). Variables with univariate association (p < 0.2) were considered for inclusion in the multivariate model, with the exception of BMI and CRP due to missing values. Peak leukocyte and neutrophil counts exhibited collinearity; only peak neutrophil count was included in the multivariate model. The parsimonious multivariate model is shown in Table 6.

### Ethics

This study was reviewed and approved with a waiver of consent by the Human Research Ethics Committee of The Prince Charles Hospital, Queensland (HREC/21/QPCH/81732).

## Results

The records of 619 patients who were admitted from February to July 2022 with COVID-19 were screened, and 190 patients were eligible for inclusion in the study (Fig 1). Follow up was complete for all patients. Diagnosis of SARS-CoV-2 infection was made by nasopharyngeal PCR testing in 91.6% of patients, with the remainder being diagnosed by rapid antigen testing. Baseline characteristics are outlined in Table 2, and were largely similar between patients with coinfection and those without. The median patient age was 75 years, and 109 (57%) were male. The most common comorbidities in the cohort were hypertension (56%), chronic heart disease (41%), chronic lung disease (32%), diabetes mellitus (25%) and chronic kidney disease (18%). There were 57 patients (30%) who fulfilled criteria for immunocompromise, the most common reason being active haematological malignancy (12% of the total cohort).

**Table 2:** Patient characteristics.

Characteristic	Coinfection (n = 23)	No-coinfection (n = 167)	Total (n = 190)	*p-*value
**Baseline and demographic**				
Age at diagnosis (years) ^a^	73 (67-78)	77 (65-85)	75 (65-84)	0.21
Male sex ^b^	13 (57)	96 (57)	109 (57)	0.55
BMI (kg/m^2^, n = 159) ^a^	28 (25-31)	27 (24-32)	27 (24-32)	0.96
Aboriginal and Torres Strait Islander descent ^b^	3 (13)	3 (2)	6 (3)	0.03
COVID-19 unvaccinated ^b^	7 (30)	37 (22)	44 (23)	0.26
**Comorbidities**				
Immunocompromise ^b^	6 (26)	51 (31)	57 (30)	0.43
Diabetes mellitus ^b^	9 (39)	39 (23)	48 (25)	0.09
Chronic lung disease ^b^	7 (30)	53 (32)	60 (32)	0.55
Hypertension ^b^	10 (44)	96 (57)	106 (56)	0.21
Chronic kidney disease ^b^	2 (9)	32 (19)	34 (18)	0.22
Chronic heart disease ^b^	10 (44)	67 (40)	77 (41)	0.76
**Clinical**				
Severe COVID-19 classification ^b^	19 (83)	87 (52)	106 (56)	<0.01
Radiologic abnormality ^b^	17 (74)	120 (72)	137 (72)	0.52
Peak C-reactive protein (mg/L, n = 144) ^a^	84 (26-199)	82 (31-123)	82 (31-137)	0.37
Peak leukocyte count (x10^9^/L) ^a^	14 (9-19)	11 (8-14)	11 (8-14)	0.03
Peak neutrophil count (x10^9^/L) ^a^	11 (8-15)	9 (6-12)	9 (6-12)	0.02

BMI, body mass index.

^a^ Values are expressed as median (interquartile range); p-value derived from Wilcoxon’s rank-sum test.

^b^ Values are expressed as number (percentage); p-value derived from Fisher’s exact test.

**Fig 2:**
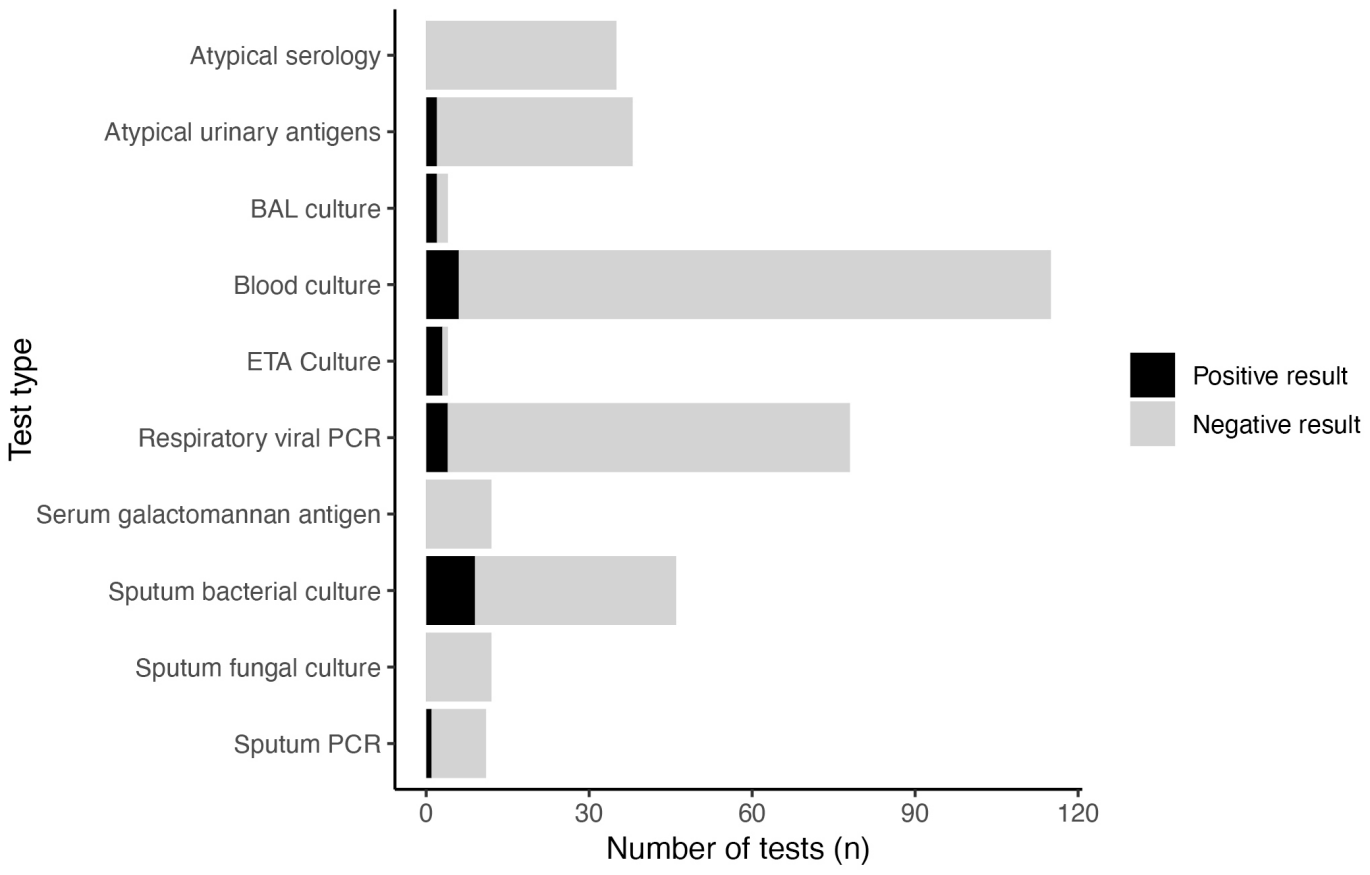
Number of microbiological tests performed in total cohort of 190 patients, stratified by test result (positive or negative). BAL, bronchoalveolar lavage; ETA, endotracheal aspirate; PCR, polymerase chain reaction.

Microbiological testing was performed in 79% of patients, and this identified 23 patients (12%) with coinfection. Of these, 5 patients were diagnosed with more than one coinfection; a common underlying factor for all five was ICU admission. In total, this signifies 28 episodes of coinfection in the patient cohort. The most common microbiological tests performed were blood cultures (61%) and respiratory viral PCR (41%). Testing for respiratory bacterial infection was less frequent, with sputum culture accounting for 24% of tests. Further breakdown of microbiological testing is displayed in Fig 2. Sixteen patients had exclusively community-acquired infections, 5 had exclusively hospital-acquired infections, and 2 had both community and hospital-acquired infections. All hospital-acquired infections were identified in patients admitted to the intensive care unit.

Bacterial infections were the most common in our cohort (5.2% of patients) and accounted for 15 episodes of coinfection (54%). Fungal organisms accounted for nine episodes (32%), and viruses for four (14%). Common organisms included *S. pneumoniae*, *P. aeruginosa*, and Influenza A (Table 3). Community-acquired infections were typified by *S. pneumoniae*, *P. aeruginosa*, *Escherichia coli*, *Klebsiella pneumoniae*, and viral infections, whereas hospital-acquired infections were characterised by *P. aeruginosa*, *Stenotrophomonas maltophilia*, *Enterobacter cloacae*, *Achromobacter xylosoxidans,* *K. pneumoniae,* and fungal organisms (Table 3). Of the 4 patients with *P. aeruginosa* infection, 2 had chronic lung disease and 1 was an intensive care patient. *Aspergillus* spp. were isolated in 2 patients. The first patient had chronic lung disease and isolated *A. fumigatus;* this was not treated as was considered clinically insignificant. The second patient was mechanically ventilated and isolated *A. niger*; which was treated as invasive pulmonary aspergillosis with voriconazole. *Pneumocystis jirovecii* infection was diagnosed in one patient with underlying malignancy and immunosuppression, and was treated with trimethoprim/sulfamethoxazole. *Candida* species were isolated frequently but none were treated with antifungal agents. It is possible that these isolates may represent infection or colonisation. Two bacterial infections were non-respiratory source bloodstream infections. Further microbiological results are described in Table 3.

**Table 3:** Microbiology of coinfection.

Pathogen	Episodes of coinfection ^a^
**Bacterial**	**15 (54%)**
*Streptococcus pneumoniae*	4
*Pseudomonas aeruginosa*	4
*Klebsiella pneumoniae*	2
Other/polymicrobial gram-negative bacilli ^b^	3
Non-respiratory source bloodstream infections ^c^	2
**Fungal**	**9 (32%)**
*Candida* species	6
*Aspergillus* species	2
*Pneumocystis jirovecii*	1
**Viral**	**4 (14%)**
Influenza A	3
Respiratory syncytial virus	1
**Total**	**28 (100%)**

^a^ Values are expressed as number (percentage)

^b^ Other gram-negative bacilli include: *Escherichia coli*, *Stenotrophomonas maltophilia*, *Enterobacter cloacae*, and *Achromobacter xylosoxidans*. One patient had a polymicrobial gram negative ventilator-associated pneumonia.

^c^ Non-respiratory source bloodstream infections were caused by *Enterococcus faecalis* and *E. coli* (both urinary sources).

Antibiotics were prescribed for 140 patients (74%), and were given empirically within 24 hours of admission in 126 patients. There was no difference between antibiotic use in the coinfection group and the no-coinfection group (Table 4). Most patients were prescribed a standard antibiotic course for pneumonia, rather than discontinuation following negative microbiology. The median duration of antibiotic use was 6 days (IQR 4−8 days). Common antibiotics prescribed were penicillins, cephalosporins, tetracyclines and macrolides. Antifungals were prescribed for one patient with fungal infection. Antivirals such as remdesivir, nirmatrelvir/ritonavir, and molnupiravir were prescribed in 119 patients (63%), and systemic corticosteroids were administered in 146 patients (77%). There was no difference in corticosteroid use between the coinfection and no-coinfection groups (87% vs 75%; *p* = 0.29). Patients in the ICU had greater corticosteroid exposure compared to those admitted to general wards (96% vs 73%; *p* < 0.01). Other COVID-19 therapies less frequently prescribed were Janus-kinase inhibitors (18%), inhaled corticosteroids (7%), and monoclonal antibodies against SARS-CoV-2 spike protein (3%).

**Table 4:** Antibiotic therapy and outcomes in patients with and without coinfection.

Variable	Coinfection (n = 23)	No-coinfection (n = 167)	Total (n = 190)	*p*-value
**Antibiotic therapy** ^a^	20 (87)	120 (72)	140 (74)	0.20
**Median length of stay** (days) ^b^	9 (4–19.5)	6 (3–9)	6 (3–9)	0.047
**ICU admission** ^a^	9 (39)	19 (11)	28 (15)	<0.01
**30-day mortality** ^a^	4 (17)	22 (13)	26 (14)	0.53
**90-day mortality** ^a^	5 (22)	26 (16)	31 (16)	0.55

ICU, intensive care unit.

^a^ Values are expressed as number (percentage); p-value derived from Fisher’s exact test.

^b^ Values are expressed as median (interquartile range); p-value derived from Wilcoxon’s rank-sum test.

Microbiologically diagnosed coinfection was associated with ICU admission and an extended hospital LOS (Table 4). ICU admission occurred 9 patients with coinfection (39%) and 19 patients without coinfection (11%) (*p* < 0.01). Intubation and mechanical ventilation were required for 6 patients in the coinfection group, and 3 patients in the no-coinfection group. Median hospital LOS was higher in the coinfection group (9 days; IQR: 4–19.5) compared to the no-coinfection group (6 days; IQR: 3–9; *p* = 0.047). There was no difference in 30- or 90-day mortality between the two groups.

Univariate associations with coinfection are displayed in Table 5, and included low body mass index, Aboriginal and Torres Strait Islander descent, ICU admission, peak leukocyte count, peak neutrophil count and severe COVID-19 classification. Multivariable modelling identified associations between coinfection and severe COVID-19 (OR: 4.31; 95% CI: 1.15–16.20; *p* = 0.03), ICU admission (OR: 3.39; 95% CI: 1.19–9.66; *p* = 0.02), and Aboriginal and Torres Strait Islander descent (OR: 17.19; 95% CI: 2.35–125.85; *p* < 0.01) (Table 6).

**Table 5:** Univariable (unadjusted) associations with coinfection.

Characteristic	Odds ratio	95% CI	*p-*value
**Baseline and demographic**			
Age (≥ 75 years)	0.41	0.16-1.05	0.06
Male sex	0.96	0.40-2.32	0.93
BMI (kg/m^2^)			
• 20-25	Ref		
• < 20	7.2	1.15-45.17	0.04
• 25-30	3.86	0.78-19.02	0.10
• 30+	2.57	0.50-13.16	0.26
Aboriginal and Torres Strait Islander descent	8.2	1.55-43.40	0.01
COVID-19 unvaccinated	1.54	0.59-4.02	0.38
**Comorbidities**			
Immunocompromise	0.80	0.30-2.15	0.67
Diabetes mellitus	2.1	0.85-5.25	0.11
Chronic lung disease	0.94	0.37-2.42	0.90
Hypertension	0.57	0.24-1.37	0.21
Chronic kidney disease	0.40	0.09-1.80	0.19
Chronic heart disease	1.14	0.48-2.77	0.76
**Clinical**			
Severe COVID-19 classification	4.37	1.42-13.39	0.01
Intensive care admission	5.01	1.91-13.13	<0.01
Radiologic abnormality	1.11	0.41-2.99	0.84
Peak C-reactive protein (mg/L)			
• Not recorded	Ref	Ref	
• < 20	1.43	0.29-6.97	0.66
• > 20	1.63	0.51-5.17	0.41
Peak leukocyte count (x10^9^/L)	1.05	1.01-1.10	0.03
Peak neutrophil count (x10^9^/L)	1.09	1.02-1.16	0.02

CI, confidence interval; BMI, body mass index.

**Table 6:** Adjusted associations with coinfection based on multivariable logistic regression.

Variable	Odds ratio	95% CI	*p-*value
Aboriginal and Torres Strait Islander descent	17.19	2.35-125.85	<0.01
Intensive care admission	3.39	1.19-9.66	0.02
Severe COVID-19 classification	4.31	1.15-16.20	0.03

CI, confidence interval.

## Discussion

The prevalence of microbiologically diagnosed coinfection was low (12%) in our retrospective study of 190 patients hospitalised with moderate to severe COVID-19 infection. Bacterial coinfection was diagnosed in only 5.2% of patients, and yet antibiotics were prescribed in 74%. We found that coinfection was associated with increased hospital LOS and ICU admission, but did not identify a difference in mortality.

Coinfections have been reported in 2–45% of patients with COVID-19 internationally, with such a broad range attributable to highly diverse study populations [2,7–11]. Our study included patients admitted to the general ward as well as intensive care, and we captured patients with both community and hospital-acquired infections. Common coinfecting pathogens in our study were *S. pneunomiae*, *P. aeruginosa* and Influenza A, all of which have been reported in international literature [2,5–13]. Notably, *S. aureus* and Influenza B infections were not identified in our cohort of patients, whereas these have been reported as common pathogens internationally [2,5–13]. This may be reflective of the overall low rates of Influenza infection observed during the pandemic [22,23]. All hospital-acquired infections were identified in patients admitted to the ICU, and typically included gram-negative bacteria and fungal organisms. Patients in intensive care typically have longer hospital stays, greater corticosteroid exposure and exposure to mechanical ventilation, all of which may increase the risk of hospital-acquired infection.

*Candida* spp. were frequently identified in sputum, bronchoalveolar lavage and endotracheal aspirate cultures. Although *Candida* is a common colonising organism, it has been reported in many coinfection studies in the literature [2,9,14,18]. It is possible that some of the isolates in our study are not clinically pathogenic. However, we have included *Candida* isolates in our results due to the limitations of fungal diagnostic techniques, and the emerging evidence of fungal infection complicating COVID-19. Current fungal diagnostic techniques are limited by the ability to sample the site of disease, are often time-consuming or insensitive, and there are inherent difficulties distinguishing between *Candida* species [18]. Knowledge on fungal infection complicating COVID-19 continues to grow, with *Candida, Aspergillus* and *Mucor* common pathogens reported in the literature [14,18]. While interest has been primarily directed at invasive fungal infections, milder fungal infections remain of relevance as a port-of-entry to more severe infections [18]. We have included all *Candida* culture results as knowledge on fungal coinfections continues to expand, however results should be interpreted with caution as colonising organisms may be included. Further studies on fungal diagnostics and coinfecting mechanisms are required to definitively distinguish between fungal infection and colonisation.

It is important to acknowledge that whilst testing rates for coinfection were relatively high at 79%, this was largely driven by blood cultures and respiratory viral PCR tests (Fig 2). Sputum bacterial culture, atypical urinary antigens and atypical pneumonia serology were only collected in 18–24% of patients (Fig 2). It is therefore possible that some patients with coinfection were not tested for it. In our retrospective study, testing was performed as per physician preference and did not follow a standardised set of guidelines. Testing rates may have differed depending on the patient’s clinical status and location (e.g., ICU vs general ward). These testing rates therefore reflect real-world practice: barriers include staff education, cost, acuity, and patients being non-productive of sputum. Induced sputum is often considered in these cases but is made more complex by the aerosolising nature of the procedure. Current diagnostic techniques are poor and further research is required to improve testing rates and yield, including incorporating newer molecular techniques and next-generation sequencing [24].

Antibiotics were widely prescribed, with 74% of the cohort receiving antibiotics. Indiscriminate use of antibiotics was common early in the pandemic due to large numbers of unwell patients with a novel disease and limited targeted treatment options such as antiviral therapies [5–8]. We suggest that antibiotics were used frequently in our cohort due to the high disease burden (moderate and severe COVID-19) and inclusion of ICU patients, some of whom had hospital-acquired infections. While antivirals were available in Australia at the time, these patients were presenting unwell with a novel disease, and thus widespread empirical prescribing of antibiotics was common. Antibiotics were prescribed upon admission for most patients, and at practitioner discretion due to a lack of clinical guidelines on when antibiotics should be considered for COVID-19 patients. Once commenced, antibiotics were usually continued (median duration 6 days) rather than being de-escalated earlier based on negative microbiology. We acknowledge that coinfection may have been under-recognised or under-diagnosed in this study, due to non-standardised testing rates. Even accounting for these shortcomings, it remains unlikely that antibiotics were required to treat bacterial coinfection in 74% of patients. It is clear that more research is required to identify biomarkers that indicate coinfection. This will assist in appropriate empirical prescribing and rationalisation of antibiotics. Factors that may be useful for clinical decision making include a history of immunocompromise or structural lung disease, leukocyte or neutrophil count, procalcitonin, radiological lobar pneumonia, and intensive care admission. Not all of these factors were included in the current study, and further research is required to identify risk factors for coinfection.

Rationalising antibiotic prescribing is important to mitigate the individual and public health threats of antimicrobial resistance. Emerging evidence suggests that the incidence of multi-drug resistant organisms increased during the COVID-19 pandemic [16]. We do not have available data on the incidence of multi-drug resistant organisms in our cohort, but nonetheless our data on antibiotic prescribing patterns are useful to support antimicrobial stewardship on a local level.

The relationship between coinfection and ICU admission is complex and likely bidirectional in nature. This limits our ability to draw causal inference. Patients with coinfection may be more likely to require ICU admission and a longer hospital LOS, but conversely ICU admission and longer hospital LOS are also predisposing factors for hospital-acquired infection. Disease severity, mode of ventilation, and immunomodulatory medications may further confound this relationship. We observed higher corticosteroid exposure in the ICU, which may increase the risk of superinfection [9]. Other studies have demonstrated that the duration of corticosteroid therapy in ICU is associated with hospital-acquired infection and infection with multi-drug resistant organisms [9]. ICU admission may therefore be considered a pre-existing risk factor for superinfection, as well as a marker of disease trajectory. The relationship is bidirectional and further research is required to delineate the major mechanism of this association. There was no difference in mortality between the coinfection and no-coinfection groups. Our study was potentially underpowered to detect the association between coinfection and mortality that has been demonstrated in other reported cohorts [2,7,10].

Our multivariate model identified severe COVID-19 and Aboriginal and Torres Strait Islander descent as being associated with coinfection. Severe COVID-19 is a plausible risk factor for coinfection, due to the significant inflammation, immune modulation, and long hospital (or ICU) stays associated with severe COVID-19; which all increase vulnerability to infection. Conversely, coinfection may also increase COVID-19 disease severity, which further adds to the complexity of this association. Aboriginal and Torres Strait Islander people comprise only a small number of the participants in this study, and the regression analyses demonstrated very wide confidence intervals. This association is best considered as hypothesis-generating. Aboriginal and Torres Strait Islander people are at higher risk of severe COVID-19 as they are disproportionately exposed to a variety of social and medical inequities [25]. These include higher rates of pre-existing medical conditions, multimorbidity, smoking, differential access to health services, overcrowding, inadequate housing, and food insecurity [25]. This heightened vulnerability may indeed extend to coinfections as well, but this must be investigated with further studies and within the wider context of the social inequities that impact health.

This single-centre study is limited by its retrospective design and small sample size, reflected by imprecise estimates of association with very broad confidence intervals. There was missing data for CRP and BMI which limited statistical analysis. Information on the timing of peak laboratory values was unavailable, introducing the possibility that these markers represent downstream changes rather than risk factors. Nonetheless, we propose our results be considered hypothesis-generating, providing a platform for further investigation. Larger, multicentre studies with sufficient power are required to establish risk factors for coinfection, diagnostic tests able to discriminate between respiratory infections, and tools for improved antimicrobial stewardship.

## Conclusion

COVID-19 was infrequently complicated by microbiologically proven bacterial coinfection, but antibiotics were widely prescribed. Our results highlight an opportunity to improve diagnostic testing and antimicrobial stewardship. Complex and likely bidirectional relationships exist between coinfection, COVID-19 severity, LOS and ICU admission. Future studies identifying risk factors and prevention of coinfection are warranted to improve the care of hospitalized COVID-19 patients.

## Supporting information

S1 File Supplementary dataset. (XLSX)

## Abbreviations

COVID-19: Coronavirus disease 2019
ICU: Intensive care unit
LOS: Length of stay
PCR: Polymerase chain reaction
SARS-CoV-2: Severe acute respiratory syndrome coronavirus 2
